# Tuberous Sclerosis Complex: A Case Series from a Romanian Genetics Center and a Review of the Literature

**DOI:** 10.3390/jcm14092974

**Published:** 2025-04-25

**Authors:** Aurora Alexandra Jurca, Ramona Hodisan, Alexandru Daniel Jurca, Emilia Severin, Sanziana Jurca, Ana Trandafir, Tiberia Ilias, Cosmin Vesa, Claudia Maria Jurca

**Affiliations:** 1 Doctoral School of Biological and Biomedical Sciences, University of Oradea, 410087 Oradea, Romania; jurca.auroraalexandra@student.uoradea.ro (A.A.J.); trandafir.analucretia@student.uoradea.ro (A.T.); 2 Department of Preclinical Disciplines, Faculty of Medicine and Pharmacy, University of Oradea, 410087 Oradea, Romaniacosmin.vesa@csud.uoradea.ro (C.V.); maria.jurca@didactic.uoradea.ro (C.M.J.); 3 Department of Genetics, University of Medicine and Pharmacy “Carol Davila” Bucharest, 020021 București, Romania; 4 Faculty of Medicine and Pharmacy, University of Oradea, 410087 Oradea, Romania; jurca.sanzianaiulia@student.uoradea.ro; 5 Department of Medical Disciplines, Faculty of Medicine and Pharmacy, University of Oradea, 410087 Oradea, Romania; ioana.ilias@didactic.uoradea.ro; 6 Regional Center of Medical Genetics Bihor, County Emergency Clinical Hospital Oradea (Part of ERN ITHACA), 410469 Oradea, Romania

**Keywords:** tuberous sclerosis, cortical and subcortical nodules, *TSC1* gene, *TSC2* gene

## Abstract

**Introduction**: Tuberous sclerosis complex (TSC) is a rare multisystemic genetic disorder characterized by the formation of benign tumors in various organs, including the central nervous system, skin, kidneys, and heart. The diagnosis is based on well-defined clinical criteria, such as those from Schwartz (2007) updated in 2012 by the International Tuberous Sclerosis Complex Consensus Group. The study aims to investigate the clinical, imaging, and molecular characteristics of patients diagnosed with tuberous sclerosis and to explore the correlation between specific genetic mutations (*TSC1* and *TSC2* genes) and the severity of clinical manifestations. **Material and Methods**: This is a retrospective longitudinal study of 13 patients diagnosed with tuberous sclerosis, identified in the records of the Bihor Regional Center for Medical Genetics (BRCMG) within the Bihor County Emergency Clinical Hospital from 1984 to 2024. Clinical, imaging, and molecular features were assessed. Patients were evaluated by a multidisciplinary team, including a geneticist, pediatrician, neurologist, psychiatrist, and psychologist. Clinical and imaging data were retrospectively collected from the congenital malformations and genetic disease records of BRCMG Bihor and statistically analyzed. **Results**: All patients showed clinical and imaging signs consistent with the diagnosis of tuberous sclerosis. Neurological manifestations were present in 83% of patients, including epilepsy and cognitive delays. Renal lesions were detected in 46% of cases, and dermatological lesions, such as facial angiofibromas, were observed in 69% of patients. Mutational variants identified in the *TSC2* gene correlated with a more severe clinical presentation, including severe intellectual disability and treatment-resistant seizures, compared to variants in the *TSC1* gene. **Conclusions**: Our study, although involving a small number of patients, highlights the clinical heterogeneity of tuberous sclerosis and the importance of a multidisciplinary approach in patient management. Early diagnosis and ongoing monitoring are essential to improving the quality of life for patients. Further studies are needed to assess the impact of therapeutic interventions and genetic correlations within the studied population.

## 1. Introduction

Tuberous sclerosis complex (TSC) is a rare genetic disorder inherited in an autosomal dominant manner, with an estimated prevalence ranging from 1 in 6000 to 1 in 10,000 live births [1]. It is a multisystem disease characterized by the development of hamartomas in multiple organs, including the central nervous system, skin, kidneys, heart, and lungs [2]. The primary cause of TSC is mutations in the *TSC1* (hamartin) or *TSC2* (tuberin) genes, leading to dysregulation of the mTOR signaling pathway and the formation of benign tumors [3]. TSC represents a significant global healthcare challenge due to the complexity of its clinical management and the need for continuous interdisciplinary monitoring.

The clinical presentation of tuberous sclerosis complex is highly variable, encompassing a wide range of manifestations, including seizures, developmental delays, cognitive impairment, autism, renal lesions (angiomyolipomas), cardiac tumors (rhabdomyomas), skin lesions (facial angiofibromas), and pulmonary involvement (lymphangioleiomyomatosis). Dysregulation of the mTOR pathway, caused by genetic mutations, leads to the hyperactivation of cell growth processes, which in turn explains the development of hamartomas in various organs. Early diagnosis and continuous monitoring are critical for preventing severe complications and optimizing patients’ quality of life [4,5].

Recent therapeutic advancements, such as the use of mTOR inhibitors, have provided new treatment options for controlling severe manifestations, including refractory seizures and renal damage. However, managing TSC remains complex, requiring close collaboration between geneticists, neurologists, nephrologists, and other specialists.

In Romania, care for individuals with TSC is supported through the National Program for the Treatment of Rare Diseases, implemented by ten medical units located in main cities of the country [6]. Additionally, since 2017, there are two centers of expertise specializing in pediatric neurology, which offer multidisciplinary services for the diagnosis and treatment of TSC. Despite these initiatives, there remains a data gap regarding the epidemiology and clinical manifestations of TSC in the country. Barriers to accessing specialized care and genetic testing persist, including limited access to specialized medical centers, financial constraints, insufficient awareness or training among healthcare professionals, long waiting times, and limited availability of genetic testing. These barriers underscore the need for comprehensive national studies and the development of more centers of expertise for TSC to ensure integrated medical care, research, and support services to improve the lives of individuals living with a rare disease like TSC.

**Figure 1:**
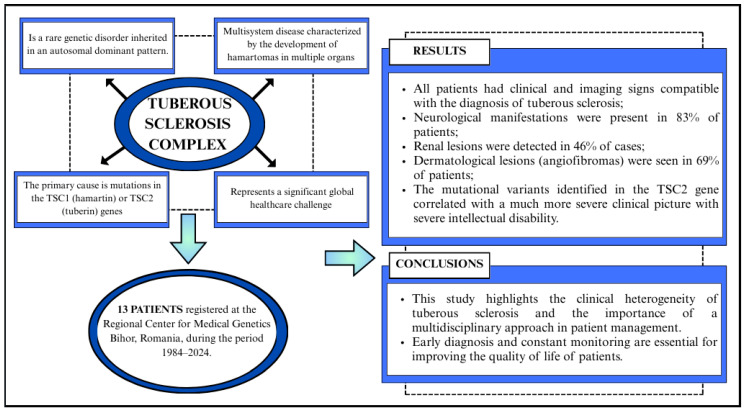
Flowchart. Clinical and diagnostic pathway of patients with tuberous sclerosis complex (TSC)–a 40-year retrospective analysis.

This paper aims to retrospectively analyze the clinical, imaging, molecular, and evolutionary characteristics of patients diagnosed with tuberous sclerosis in the records of the BRCMG over a period of 40 years (1984–2024). The results obtained will contribute to a better understanding of the disease’s impact on patients and to the development of management recommendations to improve their quality of life (Figure 1).

## 2. Materials and Methods

This study is a retrospective longitudinal study conducted over a 40-year period (1 January 1984–31 December 2024) at the Bihor Regional Center for Medical Genetics within the Bihor County Emergency Clinical Hospital. It included patients diagnosed with tuberous sclerosis, who were evaluated by a multidisciplinary team consisting of geneticists, pediatricians, neurologists, psychiatrists, cardiologists, dermatologists, psychologists, dentists, and orthopedists. Key indicators include the presence of hamartomas in several organs (brain, skin, heart, kidneys) as well as mutations in the TSC1 and TSC2 genes.

A total of 13 patients (5 men and 8 women) were included, all diagnosed with tuberous sclerosis according to the Schwartz 2007 clinical criteria updated in 2012. Clinicopathological data, including demographic, clinical, imaging, and molecular information, were collected from the genetic diseases and congenital malformations records of the BRCMG and correlated for analysis.

Written informed consent was obtained from the mother prior to participation in the study. DNA was extracted from peripheral blood lymphocytes of the patient using standard extraction procedures. The patient was tested using next-generation sequencing (NGS) with a multi-gene panel of 2 genes *TSC1* and *TSC2*. The targeted regions were sequenced with ≥50× depth or were supplemented with additional analysis. After high-throughput sequencing using Illumina technology, the output reads were aligned to a reference sequence (genome build GRCh37; custom derivative of the RefSeq transcriptome) to identify the locations of exon junctions through the detection of split reads. The relative usage of exon junctions in a test specimen was assessed quantitatively and compared to the usage seen in control specimens. Abnormal exon junction usage was evaluated as evidence in the Sherloc variant interpretation framework. If an abnormal splicing pattern was predicted based on a DNA variant outside the typical reportable range, the presence of the variant was confirmed by targeted DNA sequencing. Enrichment and analysis focus on the coding sequence of the indicated transcripts, 20 bp of flanking intronic sequence, and other specific genomic regions demonstrated to be causative of disease at the time of assay design. Promoters, untranslated regions, and other non-coding regions were not otherwise interrogated. For some genes, only targeted loci were analyzed. Exonic deletions and duplications were called using an in-house algorithm that determines copy number at each target by comparing the read depth for each target in the proband sequence with both mean read-depth and read-depth distribution, obtained from a set of clinical samples. The classification of genetic variants (pathogenic, likely pathogenic, novel, or previously known) was performed according to the ACMG guidelines for genetic variant classification. Additionally, to determine the pathogenicity of these variants, the ClinVar and HGMD databases were consulted, along with relevant scientific literature providing further information on these mutations.

### 2.1. Inclusion Criteria

- Confirmed diagnosis of tuberous sclerosis according to the Schwartz 2007 clinical criteria.
- Age: Patients of all ages (children and adults) included in the BRCMG records between 1984 and 2024.
- Availability of complete clinical data: Comprehensive medical information, including family history, clinical examination, and imaging investigations (CT/MRI), and at least one follow-up evaluation after diagnosis to monitor the progression.
- Multidisciplinary evaluation: Performed by a geneticist, pediatrician, neurologist, psychiatrist, and psychologist.

### 2.2. Exclusion Criteria

- Incomplete data: Patients without sufficient medical data or clinical investigations to confirm the diagnosis of tuberous sclerosis.
- Incorrect diagnoses: Erroneous or insufficiently documented diagnoses based on the Schwartz 2007 criteria.
- Patients who could not be contacted for re-evaluation or long-term follow-up.

### 2.3. Data Collection Methods

The data for this study were collected retrospectively from the patients’ congenital malformations and genetic disease records of BRCMG. The information obtained includes:

- Detailed clinical history of each patient, including family and personal history.
- Imaging investigations: Imaging examinations (CT, MRI) to identify brain lesions and other involved organs (kidneys, heart, lungs).
- Psychological and neuropsychiatric evaluation, as well as pediatric neuropsychiatry, including information from psychological and psychiatric evaluations to determine the degree of cognitive and neurological impairment.
- Genetic evaluation. In the case of patients for whom genetic testing could be performed, the identified genetic mutational variants as well as deletions/duplications were reported.

### 2.4. Evaluation Procedure

Each patient was evaluated by a multidisciplinary team, which included:

- Geneticist: Performed genetic evaluation to identify clinical signs consistent with TSC and detect mutations in the *TSC1* or *TSC2* genes. Assessment included family history and genetic counseling.
- Pediatrician: Conducted general health assessments and monitored growth and development, identifying systemic manifestations of TSC.
- Neurologist/Pediatric Neurologist: Evaluated for neurological manifestations such as cortical tubers, subependymal nodules, subependymal giant cell astrocytomas (SEGAs), and epilepsy/seizures.
- Psychiatrist/Pediatric Psychiatrist: Assessed for TAND (TSC-associated neuropsychiatric disorders), including autism spectrum disorder (ASD), attention-deficit/hyperactivity disorder (ADHD), anxiety, depression, and aggressive behaviors.
- Dermatologist: Identified characteristic skin lesions associated with TSC, including facial angiofibromas, hypomelanotic macules, shagreen patches, and ungual fibromas.
- Cardiologist/Pediatric Cardiologist: Screened for cardiac rhabdomyomas, which are common in infants and may cause arrhythmias or obstructive symptoms.
- Ophthalmologist (preferably with expertise in pediatric or retinal disorders): Evaluated for retinal hamartomas and other ocular manifestations such as astrocytic retinal lesions.
- Psychologist: Conducted cognitive and behavioral assessments to identify developmental delays, learning difficulties, and intellectual disability, which are frequently associated with TSC.

### 2.5. Data Analysis

The collected data were statistically analyzed using descriptive methods to present the distribution of demographic, clinical, and imaging characteristics of the patients.

### 2.6. Ethical Considerations

This study was conducted in accordance with the ethics and patient confidentiality guidelines, with approval from the Ethics Committee of the Bihor County Clinical Hospital. All patients or their legal representatives provided informed consent for the use of medical data for research purposes.

## 3. Results

### 3.1. Demographic Characteristics

During the period 1984–2024, within the BRCMG, a total of 13 patients were diagnosed with tuberous sclerosis, representing approximately 0.19% of the total of 6700 patients with genetic diseases registered.

Table 1 presents the main demographic changes in the studied group. The female gender was the majority, with eight of the cases being women. The oldest patient was born in 1948, and the youngest in 2021. Regarding the parents’ age at the birth of the child, most parents are in the range of (21,31) years, and more precisely, nine mothers and nine fathers. The minimum age for fathers is 22 years, and for mothers, it is 21 years; on the other hand, the maximum age for fathers is 42, and the maximum for mothers is 46.

Familial cases were encountered in three patients, of whom the patients came from families with a known history of tuberous sclerosis (for two patients, the mother of one patient and the father of the other had TS, suggesting a hereditary component, while the other 11 were sporadic cases).

The age at diagnosis ranges from 4 months to 18 years. Most patients, seven (54%), had an age at diagnosis between 4 months and 8 years, five patients had an age at diagnosis in the range of (8,16), and only one patient was diagnosed at the age of 18 years.

The initial symptoms in most patients (92.3%) were seizures. One patient received an intrauterine diagnosis (or diagnosed prenatally) based on evidence of a cardiac rhabdomyoma and one patient presented with epileptic seizures.

### 3.2. Clinical Features

#### 3.2.1. Cardiac, Renal, Bone, and Ophthalmological Ultrasound Clinical Aspects

**Figure 2:**
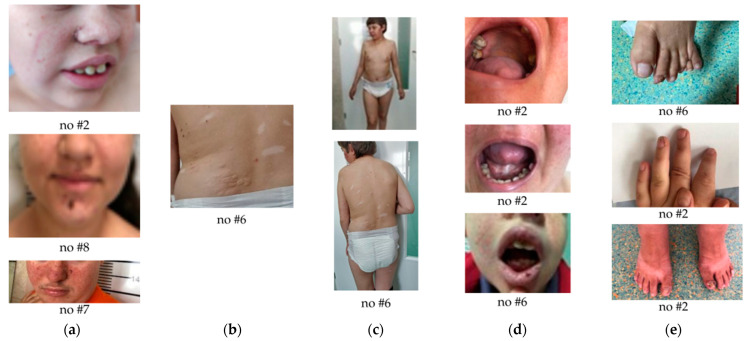
Dermatological aspects and oral cavity: (**a**) facial fibroma, (**b**) Shagreen patch, (**c**) achromic spots, (**d**) oral cavity, (**e**) ungual fibromas. Patients are labeled as no#2, no#6, etc., as shown in Table 1.

**Figure 3:**
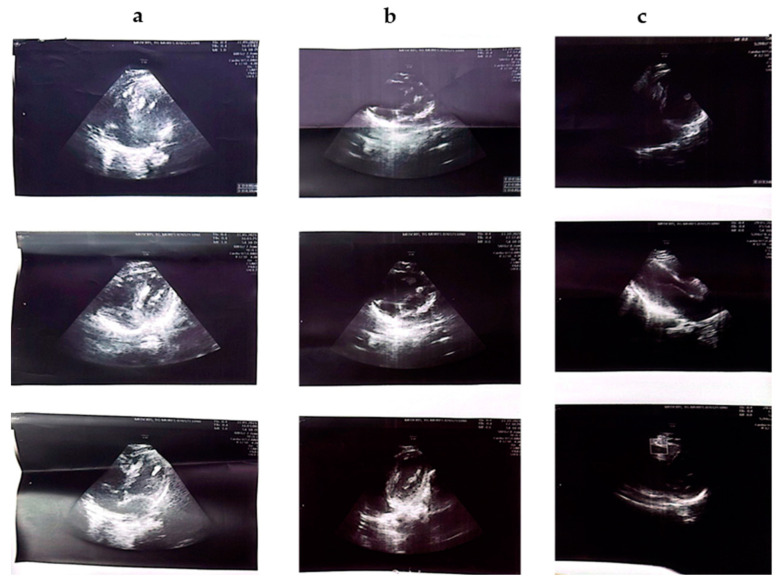
Cardiac ultrasound (no#3): ((**a**), **left**) 2021: multiple rhabdomyomas at the level of the left ventricular septum; ((**b**), **middle**) 2021: slightly reduced in volume; ((**c**), **right**) 2024: in regression.

The clinical aspects and cardiac, renal, bone, and ophthalmological ultrasound changes of each patient are described in detail in Table 2 and Table 3. Figure 2 shows photographs of some dermatological changes in some of the patients, and Figure 3 shows the evolutionary changes of the cardiac rhabdomyoma of patient no#3.

The most representative dermatological changes are highlighted in the figure below (Figure 2). The presence of facial angiofibromas, shagreen spots, acromas, intraoral and periungual fibromas can be observed.

In patient # 3, suspicion of TSC was raised in intrauterine life when ultrasound revealed the presence of tumor formations with the appearance of cardiac rhabdomyoma. At 4 months, the diagnosis of TSC was established. Figure 3 highlights the evolution of cardiac rhabdomyoma over time.

#### 3.2.2. Neuropsychiatric Aspects

The most relevant neuropsychiatric aspects: epilepsy, ASD, neuromotor retardation, language, and behavioral disorders, are described in Table 4. Patients no# 4, 7, and 12 presented with hetero- and auto-aggressiveness, and in addition to these, patient 4 had two suicide attempts. Most patients (*n* = 7) had IQ scores between 51 and 102. Three patients had IQs below 51, indicating significant intellectual disability. Only two patients had IQs above 100—one with an IQ of 120 who completed higher education, and another with an IQ of 110 who completed 12 grades and is currently employed.

#### 3.2.3. Imaging Characteristics

All 13 patients underwent neuroradiological brain scans, computed tomography and CT, and magnetic resonance imaging (MRI), as described in detail in Table 5. It is noteworthy that giant cell astrocytoma (SEGA) was present in only one patient (patient no. 1).

**Figure 4:**
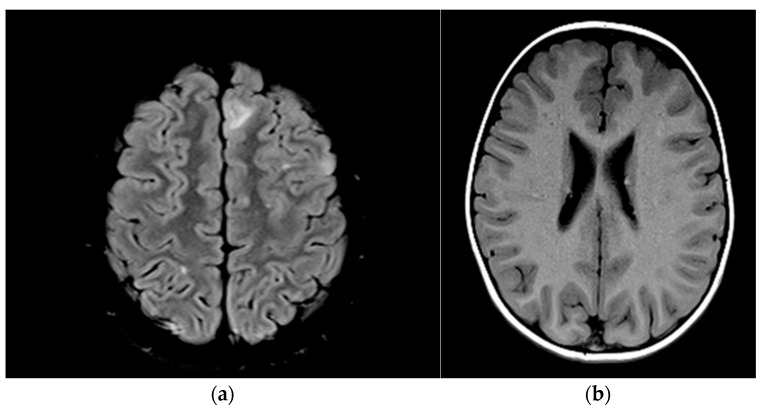
(**a**) Cortical tubers (patient no #13); (**b**) subependymal micronodules (patient no#3).

The presence of cortical tubers and subependymal nodules is highlighted by MRI images in Figure 4.

#### 3.2.4. Molecular Diagnosis

Genetic testing was successfully performed for nine patients, while it was not possible for the remaining four patients—three of whom had passed away and one who declined testing. Next-generation sequencing (Invitae Laboratories) was conducted for seven patients, while MLPA was used for the other two. The results are summarized in Table 6.

#### 3.2.5. Social, Functional, and Integration Aspects of Patient Evolution

Our study tracked the longitudinal evolution of patients with tuberous sclerosis over a 40-year period in the records of CRGM Bihor, offering a detailed perspective on the medical, social, and functional aspects of the disease (Table 7).

## 4. Discussion

The clinical manifestations of TSC are multisystemic, affecting a wide range of organs and systems, with some organs being more severely affected than others. There is no single symptom that is characteristic of the disease; rather, it presents as a constellation of signs and symptoms, which can be observed through both clinical examination and imaging [7]. The diagnosis of TSC was made based on the major and minor disease criteria [4], revised by the Tuberous Sclerosis Complex Consensus Group in 2012 [8,9,10] (Table 8).

The 13 patients with TSC were monitored over a 40-year period, with assessments of clinical, imaging, and genetic parameters.

### 4.1. Dermatological Changes

Skin changes are crucial for the clinical diagnosis of TSC and are found in many of the major disease criteria revised in 2012 [11]. Facial angiofibromas, achromic spots, and “bay leaf” spots (named after the European Mountain Ash) are present in 90% of patients [12].

Facial angiofibromas are lesions with a normal density of active melanocytes, but melanin in the epidermis is present in very low quantities. They may be present at birth or appear over time. These lesions contain adipose and connective tissue, typically located in the cheekbones, and affect 75% of patients with TSC over the age of 9 years [13].

Fibrous plaques may also appear on the forehead in 20–80% of cases. The main differences between facial angiofibromas and fibrous plaques include more pronounced vascular dilation and more significant sclerosis of collagen in angiofibromas, with hyalinization and concentric fibrosis around the follicles, leading to atrophy and compression of the follicle. With advancing age, these lesions progressively increase in size, which raises clinical concern [14].

Other skin changes include shagreen spots, found in 20% of patients, typically located in the lumbosacral region. These spots resemble collagen hamartomas, where the dermis is replaced by dense, mainly acellular hyaline collagen extending into the subcutaneous tissue.

Periungual fibromas (20–80%) have pathological characteristics similar to angiofibromas but with more intense vascularization and more compact collagen. These fibromas can penetrate the hypodermic layer, and stellate cells may also be observed. Gingival fibromas and dental enamel depressions occur in 20–50% and 90% of cases, respectively [15,16].

In our study group, facial angiofibromas were present in 62% of patients, achromic spots in 69%, shagreen patches in 46%, and nail fibromas in 38%. Two patients underwent surgical intervention for removal of these lesions. Specifically, in our cases, Figure 2 illustrates the typical dermatological signs of TSC, including facial angiofibromas, shagreen patches, hypomelanotic (achromic) macules, periungual fibromas, and intraoral fibromas. These clinical features are recognized as critical for the early diagnosis of the disease and were frequently observed in our cohort, as detailed in Table 2. For instance, patient #2 exhibited all of these features, which significantly contributed to the establishment of the diagnosis.

### 4.2. Cardiac Changes

The most characteristic changes in the heart are cardiac rhabdomyoma (cardiac rhabdomyoma). Data from the literature have demonstrated their presence in 50% of patients with TSC [17]. They often represent the first indication of the disease, and they can be diagnosed prenatally [18]. Cardiac rhabdomyomas are usually grouped in several lesions, grow over a period of time, and can reach sizes of 3 to 25 mm, most frequently being located at the level of the interventricular septum. Later, they stagnate in size and even regress by the age of 3 years. Lou et al. have shown that cardiac rhabdomyoma is found in approximately 20% of adults with TSC, but most of the time, they are asymptomatic [16,19,20]. Although benign, these tumor formations must be carefully monitored because they can lead to the appearance of arrhythmias, valvular defects, or even heart failure [20,21]. In the present study, four patients (31%) had cardiac changes; in four patients, the presence of rhabdomyoma along the right ventricular wall was evident (patient#3 and patient#6); in two patients, changes were found at the level of the mitral valve (patient#2 and patient#6). What we consider important in our case series is patient #3, who was diagnosed with TSC at the age of 4 months, after fetal ultrasound revealed cardiac rhabdomyomas. This represents a clear example of early-onset TSC, which was closely monitored over time. Figure 3 illustrates the evolution of these cardiac rhabdomyomas, which followed a typical course for this type of lesion.

### 4.3. Renal Changes

Renal involvement in TSC is most seen as angiomyolipomas (AML), renal cysts, and less commonly, oncocytomas [22]. Renal angiomyolipomas have a prevalence of 55–80% and are usually multiple and bilateral; those larger than 3 cm have an increased risk of bleeding [23]. Renal cysts are less common, occurring in approximately 14–35% of patients [22,24]. Autosomal dominant polycystic kidney disease (ADPKD) accounts for less than 2% of TSC cases [25]. The association between TSC and ADPKD phenotypes suggests a functional interaction between the genes involved, which has recently been associated with the mTOR signaling pathway [26]. Another association observed by Nair et al. is with von Hippel Lindau disease (VHL). Although TSC and VHL are caused by mutations in distinct genes, these disorders share phenotypic features and possible pathophysiological similarities. Both are autosomal dominant diseases characterized by solid and cystic renal pathologies resulting from mutations in genes that regulate tumor suppression. In both syndromes, tumors with intense vascularization occur, although RCCs are less common in TSC than in VHL. The phenotypic manifestations of these disorders occur when an inherited mutation that inactivates the *TSC1/TSC2* genes in TSC or the VHL gene in VHL syndrome is combined with an additional mutation acquired during life in the same region of the affected gene [22,27]. Most patients with AML are asymptomatic, with surgery being required only in cases of significant bleeding or suspicion of malignancy [28]. AML was present in only three patients in our study (patients no. 1, 4, and 9), while five patients exhibited an erased distinction between the cortical and medullary regions (patients #6, 7, 8, 11, and 12).

### 4.4. Other Manifestations

Bone changes in patients with TSC, especially bone cysts, were initially listed as essential conditions for diagnosis. Later, due to their rarity, they were deleted from the new clinical criteria [3]. In the oral cavity, the presence of gingival fibromas and dental depressions are part of the diagnostic criteria for tuberous sclerosis complex. Sparling et al. followed oral changes in 58 adult patients with TSC. In the studied group, gingival fibromas were present in 67% of the patients [29]. Skeletal changes were present in two patients; one presented a recurrent left knee dislocation, operated on at the age of 5 years, and the second presented a left iliac bone cyst (patients no # 5 and 7). Dental anomalies (number, size, enamel) were present in 54% of patients, and gingival fibromas in 38% of patients.

#### 4.4.1. Neurological Manifestations

##### Seizures

It represents the most common neurological symptom, sometimes being the first sign that draws attention to the diagnosis, being present in 85% of patients, underlining the major impact of this condition on the quality of life of patients with TSC [23,30]. It is the most common cause of morbidity and mortality in these patients [31,32]. Their onset can be in the first months of life, and there are various types, that can be very discreet, such as unilateral tonic, or they can be clonic, localized to the face. If sometimes, the seizures are localized at the beginning, over time, they become generalized and intractable to treatment [33]. The study by Diallo et al., carried out over a period of 12 years, included a group of 518 patients hospitalized for recurrent epileptic seizures; of these, 12 had TS [34]. In our group, most patients had seizures as their initial symptom, these being noted in 11 of the 13 patients included in the study (84.6%). For example, patient #1 presented with seizures at the age of 3 years, leading to further investigations and eventual diagnosis. In patient #6, seizures began at age 8 and were resistant to conventional antiepileptic therapy, suggesting a more severe neurological course. Interestingly, patient #7 presented both with refractory epilepsy and atypical autism, a combination known to be associated with poorer neurodevelopmental outcomes.

#### 4.4.2. Cortical and Subcortical Tubers

Cortical and subcortical tubers are the characteristic brain malformations of TSC, being present in 80–90% of cases [35]. Subependymal nodules appear along the walls of the lateral ventricles and the third ventricle, being present in a high percentage (80%) [6]. Cortical and subcortical tubers in our group were present in 77% of patients, cortical calcifications in 62%, and nodular lesions were present in 31%, this being consistent with data in the literature. In several cases, these tubers were associated with early-onset epilepsy and developmental delays, such as in patient #4, who had multiple cortical tubers and severe epilepsy with neurodevelopmental regression.

#### 4.4.3. Tuberous Sclerosis-Associated Neuropsychiatric Disorders (TANDs)

The term “TAND” and associated terminology were introduced in 2013 and are accepted as umbrella terms that encompass interrelated neuropsychiatric manifestations common in Tuberous Sclerosis Complex. These manifestations include behavioral, psychiatric, intellectual, academic, neuropsychological, and psychosocial difficulties and disorders [5,36]. Autism spectrum disorders occupy a special place, being present in approximately 25–50% of patients [37]. Van Eeghen et al. observed that ATS patients with TSC present severe, intractable epileptic seizures with worsening cognitive prognosis [38]. Behavioral disorders, such as mood swings, self-injury, obsessions, aggression, impulsivity, eating, and sleeping difficulties, may also be present. In our group, autism was present in two patients; patient #7 has an atypical form of autism associated with severe epilepsy that is difficult to control therapeutically. Behavioral disorders were identified in four patients, manifesting themselves through phenomena of self- and hetero-aggressiveness in one case, and two suicide attempts were noted. These findings, especially the presence of suicidal ideation and attempts in a pediatric TSC population, are rarely documented in similar cohorts and underline the necessity of routine neuropsychiatric screening. Severe intellectual disability was also present in four patients, all of whom carried pathogenic TSC2 mutations, reinforcing existing data about genotype–phenotype correlations. The inclusion of these neuropsychiatric extremes emphasizes the heterogeneity of TAND and the need for individualized, multidisciplinary psychosocial intervention.

### 4.5. Molecular Changes

TSC is caused by mutations in the *TSC1* and *TSC2* genes. The *TSC1* gene is located on chromosome 9 (9q34.3) and contains 23 exons. The encode region is included between exons 3–23, exon 2 being alternatively spliced [39,40]. The gene encodes a protein called hamartin which has a molecular weight of 130 kD. The *TSC2* gene is located on chromosome 6p13.3 and contains 41 exons. The encoded protein is called tuberin and has a molecular weight of 200 kD. Alternative splice sites are located at exons 25 and 31 to create isoforms 4, 5.

Molecular diagnosis of the disease is accomplished by sequencing the two genes using new-generation techniques, the most commonly used being gene panels. Multiplex ligation-dependent Probe Amplification (MLPA) is also used for CNV analysis, although new protocols also include CNV analysis in sequencing techniques (NGS) [41,42]. However, whole exome analysis is becoming increasingly accessible [43].

In the patients studied, mutational variants were identified in both *TSC* genes; three patients had mutational variants in the *TSC1* gene, and five patients in the *TSC2* gene, some of which are currently not registered in international studies (Table 6).

Despite the negative MLPA result, patient#6 was included in the study due to a clinical phenotype strongly consistent with tuberous sclerosis, meeting the 2012 Consensus Criteria [44] based on characteristic clinical and imaging findings, and acknowledging the possibility of mosaicism, which may limit the detection of pathogenic variants through standard molecular testing. Further NGS testing was recommended; however, the test was not affordable for the patient and her family.

#### 4.5.1. Comparation Between Mutations in *TSC1*, *TSC2* Gene and Exon Deletions

The mutational variants in the *TSC1* gene identified in the patients in the study, c.2347C>T and c.1270A>T, generate a premature stop codon, which interrupts the synthesis process of the hamartin protein. This leads to the complete loss of protein function. The clinical effect is like that of the frameshift mutation found in patient no. 4 (c.526dup), resulting in severe forms of TSC, with manifestations such as epilepsy, mental retardation, and benign tumors.

Mutational variants in the TSC2 gene were identified in the patients in the study. The frameshift mutation in TSC2 c.3559dup has a similar effect to nonsense and frameshift mutations in TSC1, leading to a complete loss of protein function. These are associated with severe forms of TSC, which include serious symptoms such as epilepsy and the development of benign tumors. The TSC2 gene currently has 2213 pathogenic loss-of-function variants reported. Splice site mutations affect the splicing process of messenger RNA, which can lead to the production of a partially functional protein. In this case, the symptoms are milder compared to frameshift or nonsense mutations but can still include seizures and other neurological manifestations [9,39,40]. We specify that, to our knowledge, the variants identified in patients no 4, 5, and 7 have not been described in the specialized literature. Given that these variants are previously unreported and likely pathogenic, additional experimental or bioinformatic evidence is required to confirm their impact on protein function. Further studies are necessary to evaluate how these mutations affect TSC2 protein function and contribute to the clinical presentation of patients. Georgieva et al., in a study involving 42 patients/families with TSC in Bulgaria, identified mutations in the TSC1 and TSC2 genes in 38 families. “Hotspot” mutations were found in exon 9, 15, 17, and 18 of the TSC1 gene (73% of patients) and in exons 13 and 34 of the TSC2 gene (22% of patients) [45]. In contrast, our study did not detect mutations at these loci, likely due to the smaller patient sample size. Mutations in the TSC2 gene in the patients of the study were associated with more severe forms of the disease compared to mutations in TSC1. This aligns with existing literature, which suggests that TSC2 mutations are more frequently responsible for the severe forms of the disease (51–81%) compared to TSC1 mutations (24%) [46]. This clinical variability is important to consider for proper monitoring and personalized management of each patient.

#### 4.5.2. Exon Deletions

Exon deletions in *TSC1* and *TSC2* result in the loss of important parts of the gene, resulting in truncated and nonfunctional proteins. This results in complete loss of protein function and severe forms of TSC, similar to frameshift and nonsense mutations. Clinical manifestations associated with exon deletions typically include epilepsy, mental retardation, and benign tumors, similar to those seen in complete loss-of-function mutations.

#### 4.5.3. Genotype–Phenotype Correlation

Genotype–phenotype correlations in tuberous sclerosis complex are not yet well established, although some trends have been observed in the literature. Mutational variants in the *TSC1* and *TSC2* genes result in phenotypes that may overlap. Although some phenotypes have been associated with specific mutational variants, the assessment of neurological and cognitive phenotypes is not based solely on the mutations present in the two genes. For example, mutations in the *TSC2* gene have been associated with earlier onset of epilepsy and epilepsy accompanied by intellectual deficits [47,48,49,50]. Interestingly, patients with a TSC phenotype but without an identifiable germline pathogenic variant usually present with milder systemic and neurological manifestations. In general, alterations that lead to decreased function of the tuberin protein are associated with more severe symptoms [47,48,49].

Furthermore, variants located in the flanking regions of the *TSC2* gene, as opposed to those in its central portion (exons 22–33), have been correlated with an increased risk of infantile spasms [31,51]. In contrast, patients with mutations in the *TSC1* gene tend to present symptoms associated with anxiety disorders and milder forms of autism [52]. Regarding renal manifestations, extensive deletions affecting both the *TSC2* gene and the adjacent PKD1 gene are closely linked to the occurrence of polycystic kidney disease [53,54,55].

Although it seems that patients with mutational variants in the TSC2 gene have a more severe clinical picture (early onset of seizures, severe intellectual disability, renal abnormalities, retinal depigmentation), molecular studies have not clearly established a correlation between the various genotypes, phenotypic aspects or with the severity of the disease [23,56]. Patients with mutations that inactivate the tuberin GAP domain of the *TSC2* gene have a more severe phenotype [56]. A clear genotype–phenotype link was established only in patients with the *TSC2* c.3106T>C and *TSC2* c.2714G>A mutational variants; these patients presented a moderate form of the disease and seizures [57].

In the study group, the five patients with mutations in the *TSC2* gene had a more severe clinical picture, the onset being with seizures in all five patients. In the evolution, patients no # 1, 4, 6, and 7 continue to present epileptic seizures despite all the anticonvulsant treatment instituted and associated intellectual disability. Patient no. 1 developed an intracranial tumor in the third ventricle with extension into the right lateral ventricle, for which surgery was performed in 2023. Postoperatively, she presented temporary left paresis (remitted after 3 weeks) and blindness in the right eye, being the only patient in our study series with associated tuberous lesions in the optic nerve of the right eye, and she is also the only one who presents SEGA. It is worth noting that patient no. 13 has an attenuated clinical picture, without intellectual disability, but the surprise at molecular testing was that she is the carrier of a mutational variant likely pathogenic for progressive muscular dystrophy. The mutational variant identified in this patient, c.960+2T>G, has been previously reported in the literature [58].

Our study was conducted over a long period of time—40 years—during which genetic testing has been increasingly integrated into both the private and public healthcare systems. In Romania, access to molecular testing within the public healthcare system was not available until 2014, when the Ministry of Health established, by Order, the Regional Genetics Centers. These centers, as part of a National Health Program, provide access to genetic investigations. However, not all genetic tests are covered by the National Health Insurance Fund, which prevents many families from affording the high costs of testing, leading to refusals. In 2021, through collaboration with Invitae, the opportunity for genetic testing became available, prompting a recall for reevaluation of TSC patients under follow-up who had not yet undergone genetic testing. Some patients agreed to pay for the test, while others, facing socioeconomic hardships, declined. It is merely a coincidence that the time interval between symptom onset and genetic diagnosis was shorter in patients with TSC1 mutations.

### 4.6. Treatment

Although mTOR pathway inhibitors have been used for over 10 years, treatment criteria were established at the 2012 International Consensus Conference on TSC, with refractory epilepsy being included later [59]. In our case series, mTOR inhibitors were recommended for two patients whose seizures persisted despite treatment with conventional anticonvulsants, but their parents refused the treatment. The other two patients with seizures refractory to anticonvulsants unfortunately passed away before mTOR inhibitors were approved for use. Currently, except for patients #1, 6, and 7, all other patients are no longer experiencing seizures, and those who still have seizures (e.g., patient #2) have them well controlled with anticonvulsants.

### 4.7. Social and Functional Evolution of Patients with Tuberous Sclerosis: Monitoring, Survival, and Transition to Adulthood

The evolution of patients with tuberous sclerosis in this study revealed significant variability depending on the severity of the disease manifestations. Regarding education, four patients (31%) were not educated due to a severe degree of intellectual disability; six patients (46%) are enrolled in primary education, currently two of whom are at a special school, patient#1 being permanently accompanied by his mother, and two patients (15%) have pursued higher education, completing college or university.

Occupationally, five patients (38%) managed to get a job, demonstrating a functional level sufficient for integration into the workforce. Most patients, however, were severely affected by the neurological and cognitive manifestations of the disease, which significantly restricted their possibilities of participating in social and professional life. During the follow-up period, four patients (31%) died, one of whom died at the age of 16, and three patients died in adulthood, reflecting the severity of the disease in some cases.

These data highlight the profound impact of the disease on patients’ lives, both in terms of educational and professional development and survival prognosis. Although some patients were able to achieve moderate functional levels, most experienced severe difficulties, highlighting the need for continuous monitoring and multidisciplinary care to improve quality of life.

The transition from pediatric to adult care is a significant challenge for patients with tuberous sclerosis, due to the complexity of the condition and the need for an integrative, lifelong approach. Adolescence and early adulthood are periods marked by significant changes in caregiving responsibilities and needs. The time spent in this period is often marked by worsening neurological and cognitive manifestations, as well as difficulties with social, educational, and occupational integration [60,61].

At this stage, patients require a continuous care network, which includes not only periodic monitoring of neurological and renal conditions but also support for psychosocial aspects. Integration into adult life, especially in the workplace, is a challenge, with most patients experiencing difficulties in finding or maintaining a job due to cognitive and behavioral disabilities. In this regard, adapted education and vocational training programs can facilitate their transition and integration. There are many studies in the specialized literature that emphasize the importance of this stage. Bar C et al. emphasize the persistent difficulties in coordinating and regularity of monitoring patients with TSC, even in the context of existing international guidelines. Although, in their study group of 78 patients with TSC, the majority had a positive transition, the authors noted that improvement in transition programs is needed to ensure continuity of care between the pediatric and adult health systems, especially for those with TSC, epilepsy, and significant cognitive and psychiatric impairments [62].

A holistic approach to care is essential for adults with tuberous sclerosis. This should include effective assessment and management of seizures and other associated symptoms, ongoing monitoring of physical and mental status, and psychosocial support. Early interventions, educational and vocational support, and ongoing monitoring are essential to facilitate social integration and ensure a smooth and successful transition into adulthood. Jansen et al., in a study that included 143 patients with TSC (TOSCA), aimed to analyze the impact of TSC on the health status, quality of life (QoL), and psychosocial well-being of affected individuals and their families. The authors highlighted the significant impact of TSC on education, career, and social life for both patients and their families. This effect is reflected in the quality of life, with frequent episodes of pain, discomfort, anxiety, or depression. Although families frequently access health services, the lack of well-structured care and adequate social support, along with poor transition between pediatric and adult care, exacerbates the difficulties encountered. Better coordination between educational, psychosocial, and medical support could significantly improve the lives of those affected [60,63].

The main limitation of our study is the small number of cases. However, its significance lies in the exceptionally long follow-up period of 40 years, providing a rare perspective on the evolution of tuberous sclerosis. This extended timeframe has allowed us to thoroughly analyze the variability of clinical manifestations, disease progression, and the impact of different therapeutic approaches implemented over the decades.

As most current studies focus on shorter monitoring periods, this longitudinal analysis offers valuable insights into the long-term understanding of the disease and the optimization of multidisciplinary patient care.

It is important to consider that the population of Bihor County was 551,297 at the last census (2021); the 13 cases included in our study represent all known and registered cases in the county. Consequently, the disease prevalence is 0.0024%, or approximately 1 in 42,000 births, which is significantly lower than the global prevalence.

## 5. Conclusions

The study highlights the importance of multidisciplinary management and continuous monitoring to prevent complications and improve the quality of life of patients with tuberous sclerosis. Although the small number of patients is a limitation, the extended follow-up of 40 years provides valuable information about the evolution of the disease. The results emphasize the need for future studies and the development of a national registry to optimize the care of this rare condition. Furthermore, the development of Centers of Expertise for TSC, along with collaboration with European Reference Networks like ERN Ithaca, epiCare ERN, ERN NMD, and ERN ERKNet—particularly in conditions that overlap with the clinical manifestations of tuberous sclerosis—will contribute to improving patient care, advancing research, and enhancing outcomes for individuals living with TSC.

## Figures and Tables

**Table 1:** Demographic data.

Patient No.	Sex	Years of Births	Parents’ Age at Birth		Familial = F Sporadic = S	Age at Diagnosis (Months/Years)	Onset Symptoms	OBS
			Father	Mother				
01	female	2010	29	22	S	3 y	Seizures	-
02	female	1989	40	32	S	18 y	Seizures	type II diabetes, herniated disc
03	male	2021	36	25	S	4 m	cardiac rhabdomyoma	-
04	female	2005	29	27	F	5 y	Seizures	-
05	male	2010	34	35	S	5 y	Seizures	Left iliac bone cyst
06	female	1995	22	28	S	8 y	Seizures	-
07	male	2000	29	32	S	3 y	Seizures	Scoliosis,
08	female	2001	42	46	F	10 y	Seizures	-
09	male	1979	36	26	F	5 y	Seizures	-
10	female	1948	24	21	S	35 y	Seizures	-
11	female	1976	35	31	S	14 y	Seizures	
12	male	2007	31	28	S	11 y	Seizures	-
13	female	1997	27	22	S	9–10 y	Seizures	-

**Table 2:** Clinical features.

Patient No.	Facial Angiofibromas	Achromic Spots	Shagreen Patch	Gingival Fibroma	Dental Anomalies	Nail Fibroids
01	+	+	-	-	-	-
02	+	+	+	+	+	+
03	-	-	-	-	-	-
04	+	+	+	+	+	-
05	+	+	+	-	-	-
06	+	+	+	+	-	+
07	+	+	-	+	+	+
08	-	+	+	-	+	-
09	+	+	-	-	-	-
10	+	+	+	-	+	-
11	+	-	+	-	-	-
12	+	+	+	+	+	+
13	+	+	+	-	+	+

**Table 3:** Cardiac, renal and ophthalmological abnormalities at ultrasound.

Patient No.	Cardiac	Renal	Ophthalmology
01	Interventricular septal rhabdomyoma	Bilateral renal angiomyolipomas	Tuberous lesions of the right eye, optic nerve, pale, atrophic
02	Mitral insufficiency	-	-
03	multiple fetal cardiac rhabdomyomas of the ventricular septum	-	-
04	-	Bilateral renal angiomyolipomas	-
05	Hyperechoic SIV, Mitral valve with myxomatous appearance, aberrant chordae	-	-
06	Mitral valve prolapse, mild mitral regurgitation, nodular formation in the RV wall resembling rhabdomyoma	hypertrophic kidneys, polylobate contour, disappearance of corticomedullary differentiation	-
07	-	hypertrophic kidneys, disappearance of corticomedullary differentiation	-
08	-	-	-
09	-	Bilateral renal angiomyolipomas	-
10	-	-	-
11	-	Both without cortical-medullary demarcation	-
12	-	Right kidney, the disappearance of corticomedullary differentiation and intramedullary microcalcifications	-
13	-	-	-

**Table 4:** Neuropsychiatric changes.

Patient No.	Seizures	ADHD	Language	NM Development	TSA	Behavior Disorders
01	+	-	-	-	-	-
02	+	-	-	-	-	-
03	-	-	-	-	-	-
04	+	-	-	+	-	+
05	+	-	-	-	-	-
06	+	-	+	+	+	+
07	+	-	+	+	-	+
08	+	-	-	-	-	-
09	+	-	-	-	-	-
10	-	-	-	-	-	+
11	+		+	+	-	-
12	+	+	+	+	-	+
13	+	-	-	-	-	-

**Table 5:** Imagistic results.

Patient No.	CT Scan	Brain MRI
01	-	2023: Inhomogeneous tumor formation in the third ventricle and a calcified formation in the lateral wall of the right lateral ventricle, frontoparietooccipital cortico-subcortical tubers. Secondary hydrocephalus. 2023: cortico-subcortical tubers stabilized compared to the previous examination, calcified subependymal nodules. Parietal narrow tumor formation corresponding to a giant cell astrocytoma (SEGA)
02	-	2022: four bilateral hamartomatous subependymal nodules—the largest 3 mm in diameter, partially calcified—associating cortical/subcortical tubers with frontal, temporal and occipital distribution on the right and frontal and parieto-occipital distribution on the left
03	-	2021–2022 Subependymal micronodules in the lateral ventricles; 2024. subretentorial cortico-subcortical cortical tubers
04	Nodular calcifications of the cerebral hemispheres and the left cerebellar hemisphere, diffuse osteosclerosis of the skull bones, diffuse aplasia of the skull bones, aplasia of the frontal sinus	subcortical tubers; subependymal nodules
05		supratentorial subcortical tubers, subependymal nodules with calcareous inclusions, swelling of the overlying gyrus
06	calcified subependymal tubers of the lateral ventricles, left lenticular angioma, cortico-subcortical demyelinating lesions, disseminated cerebral and cerebellar calcifications,	Nodular lesions in the walls of the lateral ventricles, areas of supratentorial paraventricular and supraventricular demyelination
07	Subependymal calcifications in the lateral ventricles max 9 mm, calcifications in the depth of the Sylvian sulcus of 14 mm, calcifications on the surface of the left cerebellum of 4 mm	Cortical and subcortical tubers, subependymal nodules, lateral ventricles, right temporal arachnoid cyst
08	Mild asymmetry of the skull, hypo/hyperdense intraparenchymal cerebral infra/supratentorial areas	Bihemispheric cortical tubers cortico-subcortical fronto-temporo-parietal Calcified subependymal and ventrolateral micronodules
09	Subependymal calcifications of the lateral ventricle	-
10	Bilateral parietal and temporal cortical tubers	-
11	-	Fronto-parieto-temporal cortical tubers, perinatal hypoxic leukoencephalopathy with multiple areas of ischemic gliosis
12	-	Bihemispheric F-P-T cortical tubers Periventricular leukomalacia, corpus callosum hypoplasia, Bilateral occipital polymicrogyria
13	-	Subependymal calcareous nodules and subcortical tubers; cortical tubers, photo-calcified nodular lesions, bilateral subependymar and cortical tubers, calcified periventricular and cortical tubers (Figure 3a,b)

**Table 6:** Genetic mutation profile.

No.	Gene	Technique	NM Number	Transcript	Variant	Zygosity	Clasificaton
01	*TSC2*	MLPA		Deletion of exon 41	structural variant	heterozygous	likely pathogenic
02	*TSC1*	NGS	NM_000368.4:c.2	347C > T	nonsense	heterozygous	pathogenic
03	*TSC1*	NGS	NM_000368.4:c.1	c.1270A > T (p. Arg424^-^)	nonsense	heterozygous	pathogenic
04	*TSC2*	NGS	NM_001318831.2	c.3466del	frameshifts	heterozygous	pathogenic
05	*TSC2*	NGS		Partial Deletion (Exon 40)	structural variant	heterozygous	likely pathogenic
06		MLPA		MLPA negative			
07	*TSC2*		NM_000548.5	c.3559dup (p. Val1187Glyfs^-^47)	frameshift	heterozygous	pathogenic
08	*TSC1*	NGS	NM_000368.4	c.5:26dup	frameshift	heterozygous	pathogenic
13	*TSC2*	NGS	NM_000548.5	c.4005 + 1G > T	splicesite	heterozygous	pathogenic
	*DMD*	NGS	NM_004006.3	c.960 + 2T > G	splice site	unknown	likely pathogenic

**Table 7:** Educational, professional evolution and survival of patients with tuberous sclerosis.

No.		No Education	Kindergarten	Special Class	Primary School	High School	Faculty	Employee	Age of Death (Years)
01	FR	-	-	-	+	-	-	-	-
02	VA	-	-	+	-	-	-	-	-
03	BG	-	+	-	-	-	-	-	-
04	PO	+	-	-	-	-	-	-	-
05	BA	-	-	-	+	-	-	-	-
06	BI	+	-	-	-	-	-	-	-
07	HR	+	-	-	-	-	-	-	-
08	MC	-	-	-	-	+	-	+	-
09	BSt	-	-	-	+	-	-	+	48
10	BO	-	-	+	-	-	-	+	52
11	DN	-	-	-	+	-	-	+	40
12	DR	+	-	-	-	-	-	-	16
13	VL	-	-	-	-	-	+	+	-

**Table 8:** Major and minor criteria for TSC.

Major Criteria (11)	Observation
Depigmented macules with a 3–5 mm diameter Facial angiofibromas (over 3) Ungual fibromas (over 2) Shagreen patch Retinal hamartomas Cortical dysplasia Astrocytomas and subependymal nodules Cardiac rhabdomyomas Lymphangioleiomyomatosis renal angiomyolipomas	Genetic diagnosis: For a positive diagnosis, it is sufficient to identify a pathogenic variant in one of the two genes
**Minor criteria (7)**	
“Confetti” skin lesions Intraoral fibromas Changes in tooth enamel White patches on the retina Renal cysts Other hamartomas Sclerotic bone changes	

## Data Availability

All data are contained within the article.
